# Effects of Xylo-Oligosaccharide on the Gut Microbiota of Patients With Ulcerative Colitis in Clinical Remission

**DOI:** 10.3389/fnut.2021.778542

**Published:** 2021-12-28

**Authors:** Zongwei Li, Zhengpeng Li, Liying Zhu, Ning Dai, Gang Sun, Lihua Peng, Xin Wang, Yunsheng Yang

**Affiliations:** ^1^Microbiota Division, Department of Gastroenterology and Hepatology, The First Medical Center, Chinese People's Liberation Army (PLA) General Hospital, Beijing, China; ^2^State Key Laboratory for Managing Biotic and Chemical Threats to the Quality and Safety of Agro-Products, Institute of Food Research, Zhejiang Academy of Agricultural Sciences, Hangzhou, China; ^3^Department of Gastroenterology, Sir Run Run Shaw Hospital, Hangzhou, China

**Keywords:** ulcerative colitis, xylo-oligosaccharides, *in vitro* fermentation, gut microbiota, 16S rDNA sequencing

## Abstract

Gut microbiota dysbiosis is closely associated with ulcerative colitis (UC). Prebiotic therapy is a potential approach for UC management especially remission maintaining. Xylo-oligosaccharide (XOS) is an efficient prebiotic with proven health benefits and few side effects. However, the effects of XOS on the gut microbiota of patients with UC have not been investigated previously. The aim of this study was to evaluate the prebiotic effects of XOS on the fecal microbiota of patients with UC in clinical remission using an *in vitro* fermentation model. Five patients with UC in clinical remission and five healthy volunteers were enrolled in this study. Fresh fecal samples of UC patients were diluted and inoculated in yeast extract, casitone and fatty acid (YCFA) medium alone or with XOS. After fermentation for 48 h, samples were collected for 16S rDNA sequencing to investigate the gut microbiota composition. Differences in the gut microbiota between healthy volunteers and UC patients in clinical remission were detected using original fecal samples. Subsequently, the differences between the YCFA medium alone or with XOS samples were analyzed to illustrate the effects of XOS on the gut microbiota of UC patients. In both principal coordinate analysis (PCoA) and principal component analysis (PCA), the fecal samples of UC patients differed from those of healthy volunteers. Linear discriminant analysis effect size (LEfSe) analysis revealed that the relative abundances of *g_Roseburia* and *g_Lachnospiraceae_ND3007_group* were higher in healthy volunteers than in UC patients, while *o_Lactobacillales* abundance showed the opposite trend (*P* < 0.05). Wilcoxon rank-sum test bar plot showed that the abundances of *g_Eubacterium_halli_group* and *g_Lachnospiraceae_ND3007_group* were higher in the healthy volunteers than in the UC patients (*P* < 0.05). In addition, in UC patients, the Wilcoxon rank-sum test showed that XOS fermentation promoted the growth of bacterial groups including *g_Roseburia, g_Bifidobacterium*, and *g_Lactobacillus*, which is beneficial for recovery of intestinal diseases. These results suggest that XOS can relieve dysbiosis in the feces of UC patients in clinical remission and thus represent a potential prebiotic material for maintaining remission.

## Introduction

Ulcerative colitis (UC) is a chronic disease involving recurrent colonic inflammation causing damage to the mucosa or submucosa of colon, and the occurrence of UC is increasing globally (1). Although the etiology and pathogenesis of UC are unclear, previous studies have shown that epithelial barrier integrity disruption, intestinal immunity disorders and in particular, gut microbiota dysbiosis contribute to its development and progression (2). The treatments for UC include controlling the active inflammation and maintaining remission by using amino salicylates, antibiotics, corticosteroids and immunomodulatory drugs. However, these therapies were more likely to bring long-term side effects especially in maintaining remission due to lack of low side effect drugs for long-term use (3). In recent years, modulating the dysbiosis of gut microbiota has been become a new strategy for UC management and shown a fine application prospect (4).

Prebiotics are non-digestible compounds found in many natural foods that selectively stimulate the growth and activity of one or several bacterial groups to produce beneficial effects on the host with few side effects (5). Many clinical trials have been carried out to assess the effects of administering prebiotics such as fructo-oligosaccharide (FOS) for UC treatment and limited benefits have been shown (6, 7). Besides, a review showed that the use of traditional prebiotics including FOS and inulin may be useful especially in IBD patients with low clinical activity of the disease or to maintain remission (8). However, some researches indicated that there was no significant beneficial effect for the use of prebiotics to IBD patients, which may be due to inclusion of IBD patients with high clinical activity or insufficient use of prebiotics (9). Therefore, patient selection for clinical trials is important to the research.

Xylo-oligosaccharide (XOS) is a more efficient prebiotic than traditional prebiotics with proven health benefits via adjusting gut microbiota (10). For example, *in vitro* and *in vivo* studies have shown that XOS significantly enriched bifidobacterial populations, which mitigated inflammatory diseases (11, 12). What's more, XOS with *bifidobacterium* have been proved to alleviate colitis in an animal model (13).

Thus, the aim of this study was to evaluate the prebiotic effect of XOS on the fecal microbiota of UC patients in clinical remission via an *in vitro* fermentation model (14).

## Materials and Methods

### Fecal Sample Origins

Five patients with UC in clinical remission diagnosed by colonoscopy and five healthy volunteers, aged between 18 and 60 years, were enrolled in this case-control study. The age and sex of the UC patients and healthy volunteers are matched and details are shown in Supplementary Table 1. The exclusion criteria were as follows: (1) diabetes, cancers or other systemic or serious diseases; (2) pregnancy or lactation; and (3) probiotic, prebiotic or antibiotic agent use within 4 weeks prior to fecal sample collection. Volunteers were provided informed, written consents for collection and research of fecal samples. All the procedures used in the present study were approved by the Ethics Committee of Chinese PLA General Hospital (S2016-130-01).

### Fermentation Medium

In brief, the basic growth medium (yeast extract, casitone and fatty acid; YCFA) contained the following compounds: tryptone, 10 g/L; yeast extract, 2.5 g/L; L-cysteine, 1 g/L; NaCl, 0.9 g/L; CaCl_2_·6H_2_O, 0.09 g/L; KH_2_PO_4_, 0.45 g/L; K_2_HPO_4_, 0.45 g/L; MgSO_4_·7H_2_O, 0.09 g/L; vitamin I solution, 200 mL; and hemin solution, 2 mL. The vitamin I solution had the following components: vitamin B8, 0.05 mg/mL; vitamin B12, 0.05 mg/mL; 4-aminobenzoique acid, 0.15 mg/mL; vitamin B9, 0.25 mg/mL; and pyridoxamine, 0.75 mg/mL. The hemin solution was 1 mg/mL in 1M sodium hydroxide. XOS (Sigma Aldrich, USA) was added (8 g/L) as the sole carbon source. After adding resazurin (0.1 mg/L), an indicator of anaerobic conditions, the medium was adjusted to pH 6.5, and 5 mL was dispensed into a 10 mL bottle that was flushed with N_2_ before sterilization in an autoclave.

### Static Fermentation

Static fermentation was conducted as described previously (15). Fresh fecal samples (0.8 g) were homogenized with 8 mL of 0.1 M anaerobic phosphate-buffered saline (pH 7.0) using an automatic fecal homogenizer (Halo Biotechnology, China) to make 10% (w/v) slurries as soon as the fecal samples arrived at the laboratory.

In addition, 0.5 mL of the fecal slurry was inoculated into a sterilization bottle of 5 mL growth medium and subjected to anaerobic fermentation at 37°C. After 48 h of fermentation, the broth was centrifuged. The precipitate of the broth were stored at −80°C for DNA extraction (16). The original feces of UC patients and healthy volunteers were defined as samples of the U_FAE and N_FAE groups, while the fecal fermentation precipitate samples of UC patients in YCFA and the XOS-containing media were defined as samples of the U_Y and U_XOS groups.

### DNA Extraction and Sequencing

Bacterial genomic DNA was extracted from all samples of four groups mentioned above using a QIAamp DNA Stool Mini Kit according to the manufacturer's instructions (Qiagen, Germany). The concentration of extracted DNA was determined by a NanoDrop 2000 (NanoDrop Technologies, USA) and confirmed by 1.0% agar gel electrophoresis. The V3-V4 region of the bacterial 16S rRNA genes was amplified using the barcoded primers 341F (5′-CCTAYGGGRBGCASCAG-3′) and 806R (5′-GGACTACNNGGGTATCTAAT-3′). Amplicons were extracted from 2% agarose gels, purified using an AxyPrep DNA Gel Extraction Kit (Axygen Biosciences, USA) according to the manufacturer's instructions and quantified using QuantiFluor™-ST (Promega, USA). Purified amplicons were pooled in equimolar amounts and paired-end sequenced (2 × 250) on an Illumina MiSeq platform according to standard protocols performed by Promegene Technology, Shenzhen, China.

### Bioinformatics and Statistical Analysis

Sequences were identified by their barcodes using the Quantitative Insights in Microbial Ecology (QIIME) 1.9.1 pipeline. Low-quality sequences were removed before further analysis. Sequences were clustered into operational taxonomic units (OTUs) with a 97% similarity cutoff by Uparse (version 7.0.1090). The OTUs were assigned to taxa using the silva138 (16s bacteria database) and data were analyzed by R package (version 3.3.1). Within-community diversity (α-diversity) indexes (the ACE, Simpson and Shannon indexes) were calculated by wilcoxon rank-sum test. Venn diagram was used to show the number of common and unique species in different groups by R. Community bar diagram and heatmap were used for exhibition of community species composition and abundance by R. β-Diversity was estimated by calculating the Bray-Curtis distance matrix with QIIME and R, and statistically examined by analysis of similarities (ANOSIM). Principal component analysis (PCA) was performed to explore the variance in microbiota composition. Principal coordinate analysis (PCoA) was conducted based on the OTU level using the R package. Statistically significant differences in the relative abundances of taxa in different groups were calculated by using the linear discriminant analysis (LDA) effect size (LEfSe) method. Taxa with LDA results >2 were considered significantly enriched. Comparison differences of species between two groups were executed by wilcoxon rank-sum test in the stats package of R (17).

## Results

### Sequencing Data

**Figure 1:**
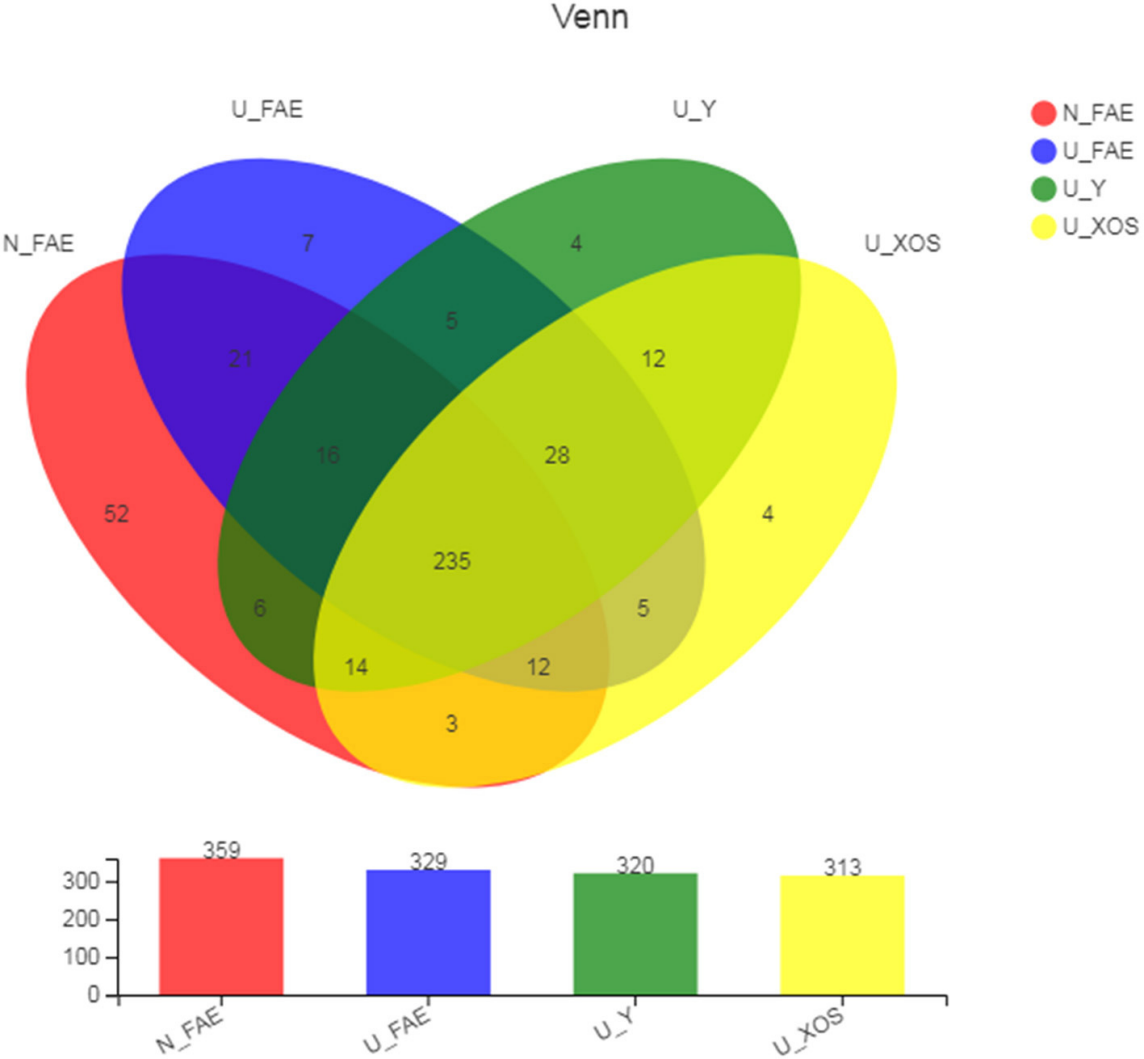
Common and unique OTUs of four groups in the Venn diagram. The OTUs were assigned to taxa using the silva138 (16s bacteria database). N_FAE: original fecal samples of healthy volunteers, *n* = 5; U_FAE: original fecal samples of UC patients, *n* = 5; U_Y: fecal fermentation samples in YCFA of UC patients, *n* = 5; U_XOS: fecal fermentation samples in YCFA+XOS of UC patients, *n* = 5.

After quality filtering, trimming and data annotation, totals of 359 OTUs and 329 OTUs were identified in the N_FAE and U_FAE groups, respectively, while 320 and 313 OTUs were found in the U_Y and U_XOS groups, respectively. A total of 235 core OTUs were detected in all four groups (Figure 1). We also found that the N_FAE group had the most unique species (52 species), with 7, 4 and 4 unique species in U_FAE, U_Y and U_XOS groups, respectively. Most species in the gut microbiota of UC patients and healthy volunteers could be cultured by static fermentation, which meant that this *in vitro* simulation system of the gut microbiota was practicable.

### α-Diversity Analysis

**Figure 2:**
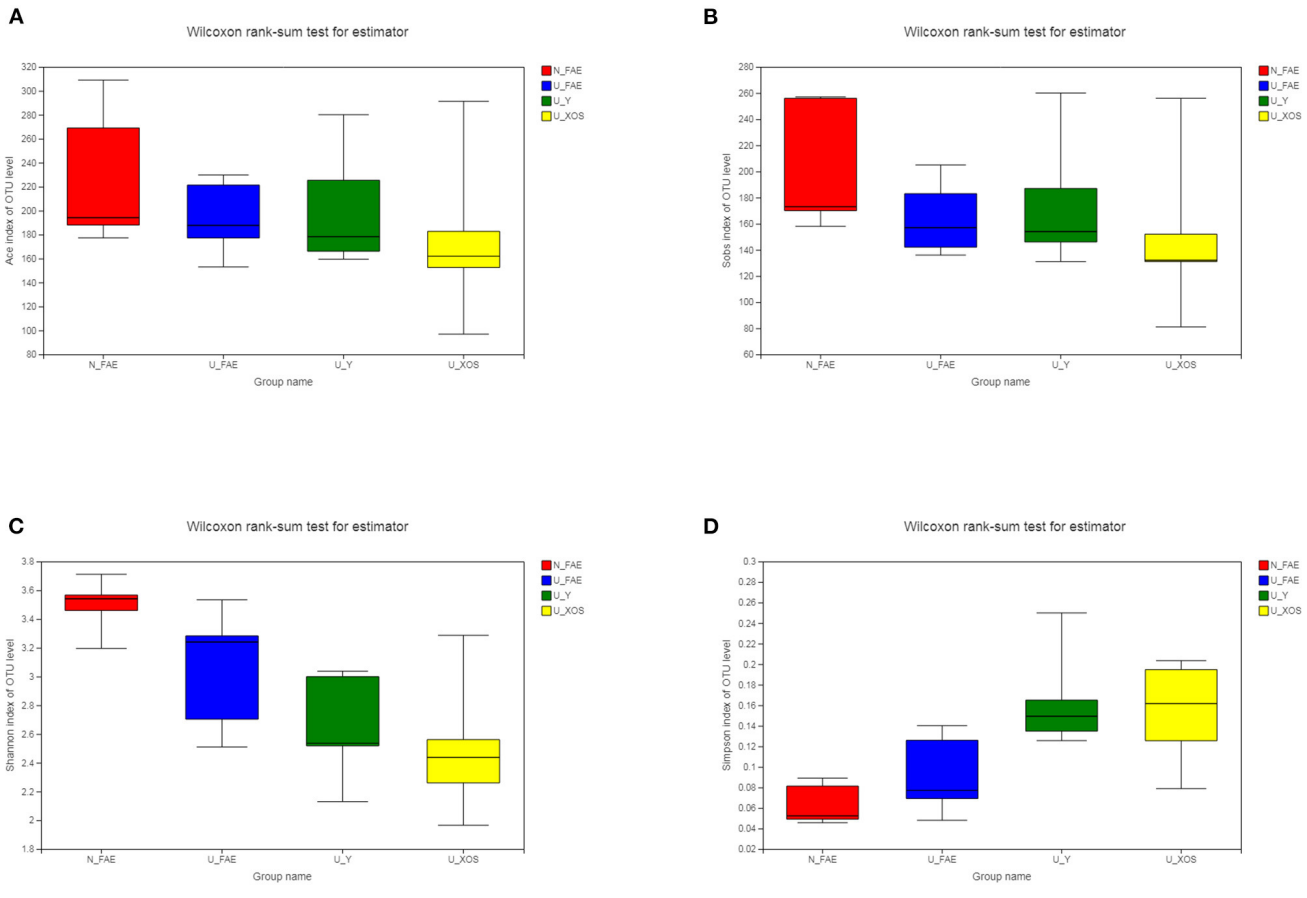
α-Diversity of the four groups. ACE index **(A)**, Sobs index **(B)**, Shannon index **(C)**, and Simpson index **(D)** in patients with UC in remission were lower than those in healthy volunteers, but these differences were not significant.

Species richness and diversity can be evaluated by measuring α-diversity, for which four indexes are commonly used: ACE and Sobs represent species richness indexes, while Shannon and Simpson represent species diversity indexes. We found that all four indexes in patients with UC in remission were lower than those in healthy volunteers, but these differences were not significant (Figure 2). Moreover, α-diversity decreased after fermentation, especially in the U_XOS group (although again, this was not significant, *P* > 0.05). This result revealed that growth of some bacteria may be inhibited by fermentation, while some bacteria showed excessive growth, especially with XOS. In general, XOS fermentation could not ameliorate the reduced α-diversity of the gut microbiota in patients with UC.

### β-Diversity Analysis

**Figure 3:**
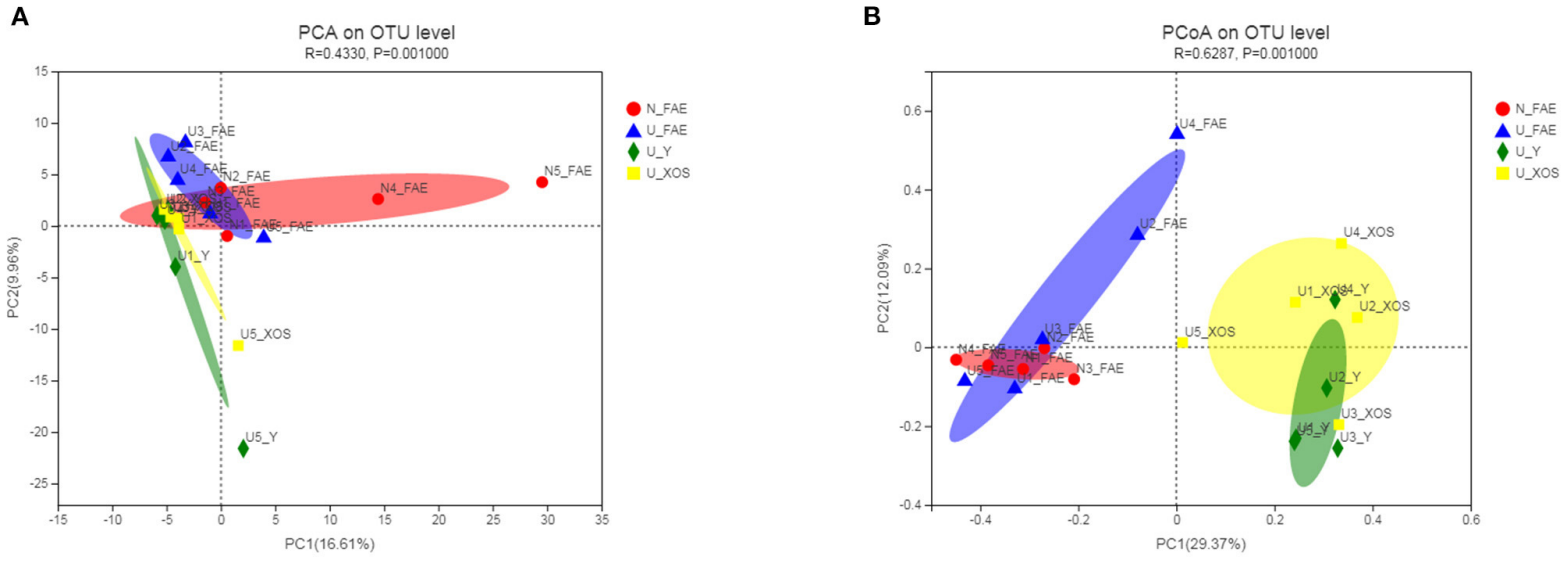
β-diversity of the four groups. According to PCA **(A)**, the four groups showed obvious separation and a similar trend was observed in the PCoA **(B)**.

β-diversity analysis was performed by calculating the Bray-Curtis distance matrix to reveal differences among the four groups, the significance of which was examined by ANOSIM. According to PCA, the four groups showed obvious separation, and the *P*-value determined by ANOSIM was 0.001, which meant that the differences among the four groups were statistically significant (Figure 3). More importantly, a similar trend was observed in the PCoA. Analysis of all samples indicated that fermentation had a significant impact on the microbiota composition, as the two FAE groups and two fermentation groups exhibited marked separation.

### Community Bar Plot Analysis

**Figure 4:**
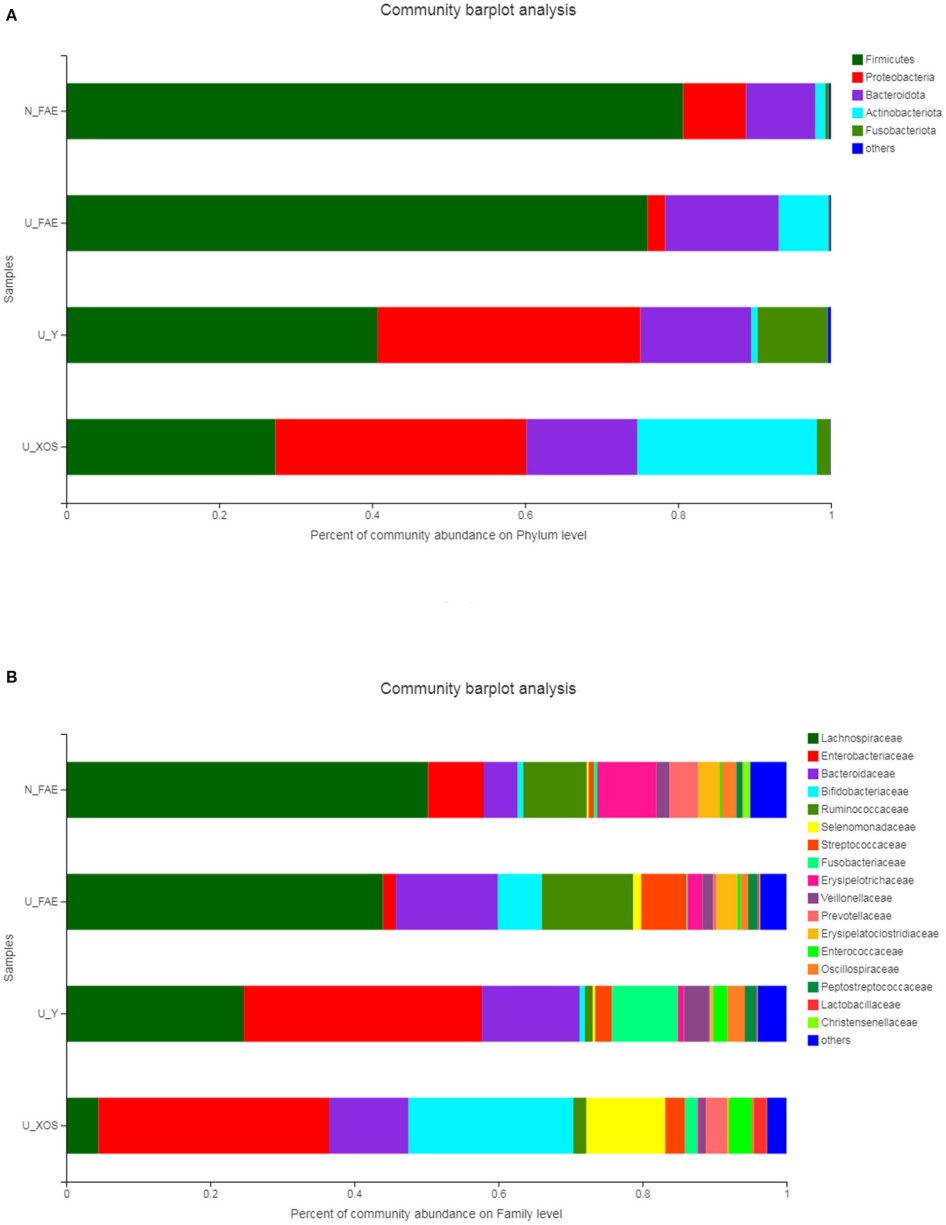
Community bar plot analysis of the four groups. **(A)** Microbiota composition at the phylum level; **(B)** microbiota composition at the family level.

Community bar plot analysis was carried out to investigate the microbiota composition in the four groups at the phylum and family levels. At the phylum level, the N_FAE samples were found to contain more *Actinobacteria* and less *Proteobacteria* than the U_FAE group. In addition, more *Actinobacteria* and less *Fusobacteria* were detected in the U_XOS group than in the U_Y group (Figure 4). At the family level, the relative abundances of *Bacteroidaceae* and *Bifidobacteriaceae* were higher, whereas that of *Enterobacteriaceae* was lower, in the U_FAE group than in the N_FAE group. Furthermore, the U_XOS group differed from the U_Y group in having increased abundances of *Bifidobacteriaceae* and *Selenomonadaceae*, and having decreased abundances of *Lachnospiraceae* and *Fusobacteriaceae* (Figure 4).

### Identification of Differentially Abundant Taxa

**Figure 5:**
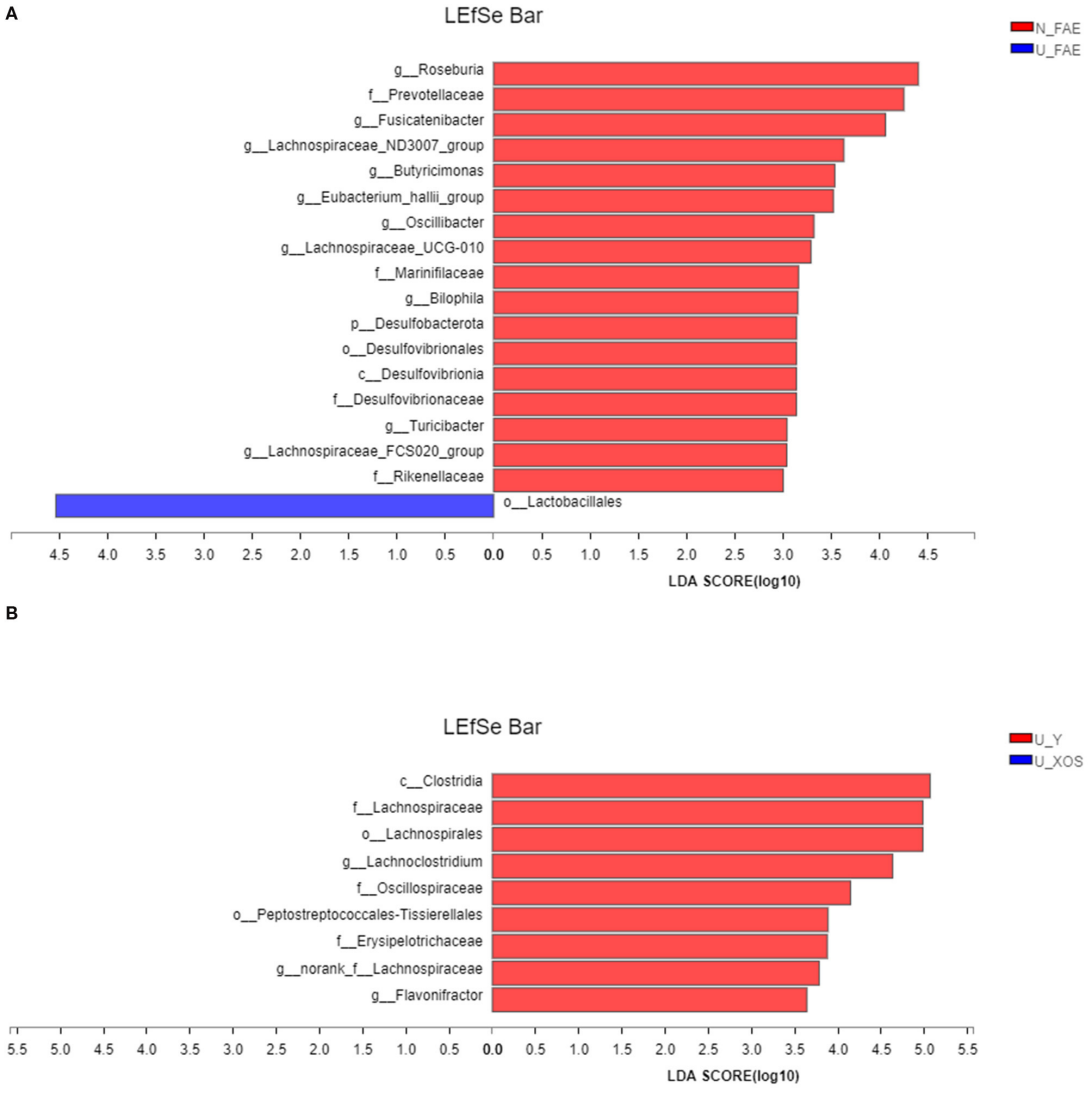
LEfSe analysis to identify the differentially abundant taxa. **(A)** N_FAE group vs. U_FAE group; **(B)** U_Y group vs. U_XOS group.

**Figure 6:**
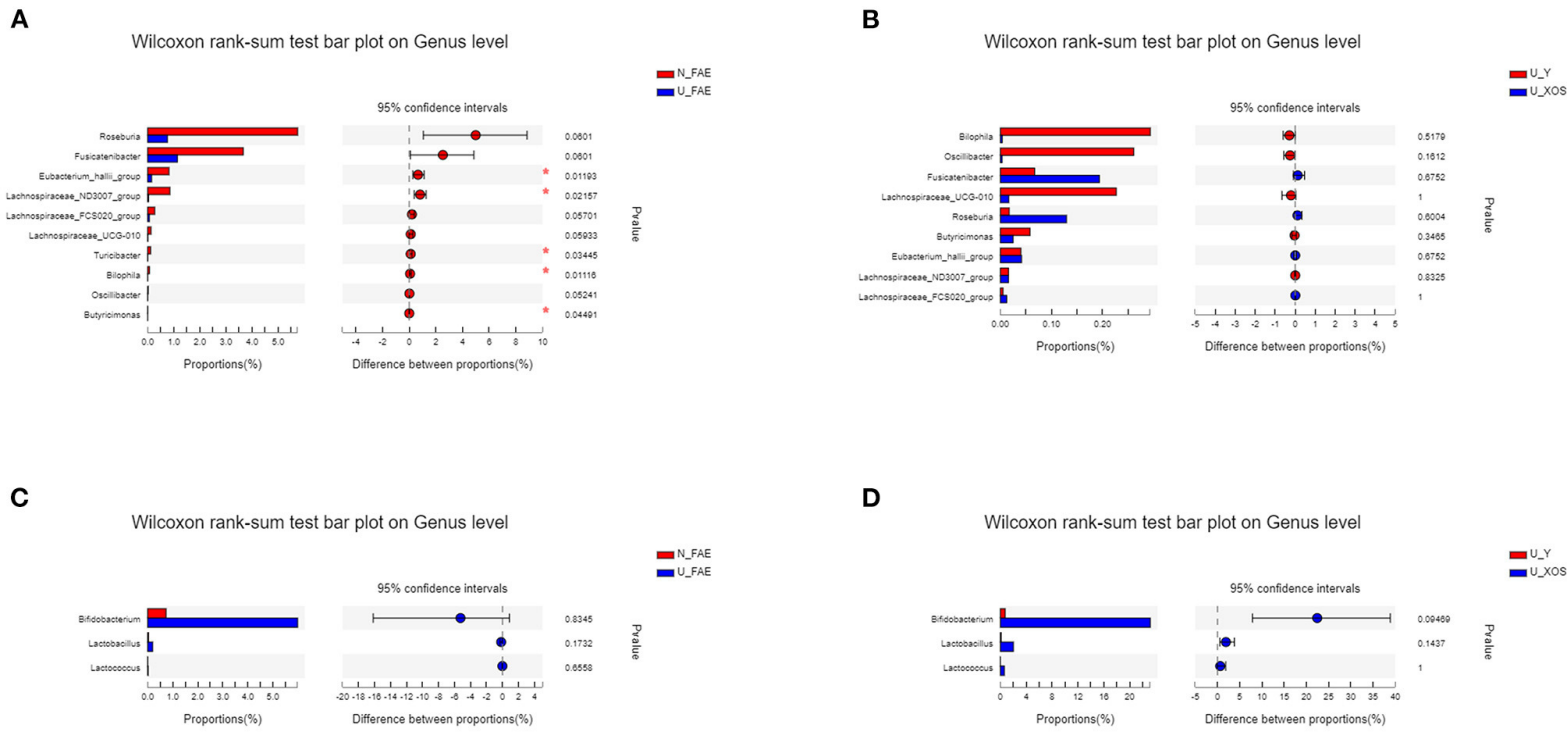
Differential abundance analysis to identify the differentially abundant taxa by the wilcoxon rank-sum test. **(A)** N_FAE group vs. U_FAE group; **(B)** U_Y group vs U_XOS group; **(C)** proportions of *Bifidobacterium, Lactobacillus* and *Lactobacillus* in the two original fecal groups; **(D)** proportions of *Bifidobacterium, Lactobacillus* and *Lactobacillus* in the two fermentation groups.

Next, LEfSe analysis was used to identify the differentially abundant taxa between the N_FAE and U_FAE groups. The results showed that the relative abundances of *g_Roseburia, g_Fusicatenibacter, g_Lachnospiraceae_ND3007_group, g_Butyricimonas, g_Eubacterium_halli_group, g_Oscillibacter, g_Lachnospiraceae_UCG-010, g_Bilophila, g_Turicibacter* and *g_Lachnospiraceae_FCS020_group* were higher in the N_FAE group, while that of *o_Lactobacillales* was higher in the U_FAE group (Figure 5A). Subsequently, differences between the proportions of these taxa were detected by the wilcoxon rank-sum test bar plot analysis at the genus level. The abundances of *g_Eubacterium_halli_group, g_Lachnospiraceae_ND3007_group, g_Bilophila, g_Turicibacter*, and *g_Butyricimonas* were higher in the N_FAE group (*P* < 0.05), whereas the abundances of *g_Bifidobacterium, g_Lactobacillus*, and *g_Lactococcus* were higher in the U_FAE group, although the difference was not significant (Figures 6A,C). To illustrate the effects of XOS on the gut microbiota of UC patients in clinical remission, LEfSe and difference analyses between the U_Y and U_XOS groups were executed. LEfSe analysis showed that the abundances of *g_Lachnoclostridium, g_norank_f_Lachnospiraceae*, and *g_Flavonifractor* were higher in the U_Y group than in the U_XOS group (Figure 5B). A wilcoxon rank-sum test bar plot at the genus level showed that *g_Roseburia, g_Fusicatenibacter, g_Bifidobacterium*, and *g_Lactobacillus* were more abundant, whereas *g_Oscillibacter, g_Bilophila*, and *g_Lachnospiraceae_UCG-010* were less abundant, in the U_XOS group than in the U_Y group, but the differences were not significant (Figures 6B,D). In addition, *g_Turicibacter* was not detected in the U_XOS group or U_Y group, which meant that it could not be cultured in the fermentation environment.

### Top-Species Identification and Phylogenetic Tree for Every Sample in the Four Groups

**Figure 7:**
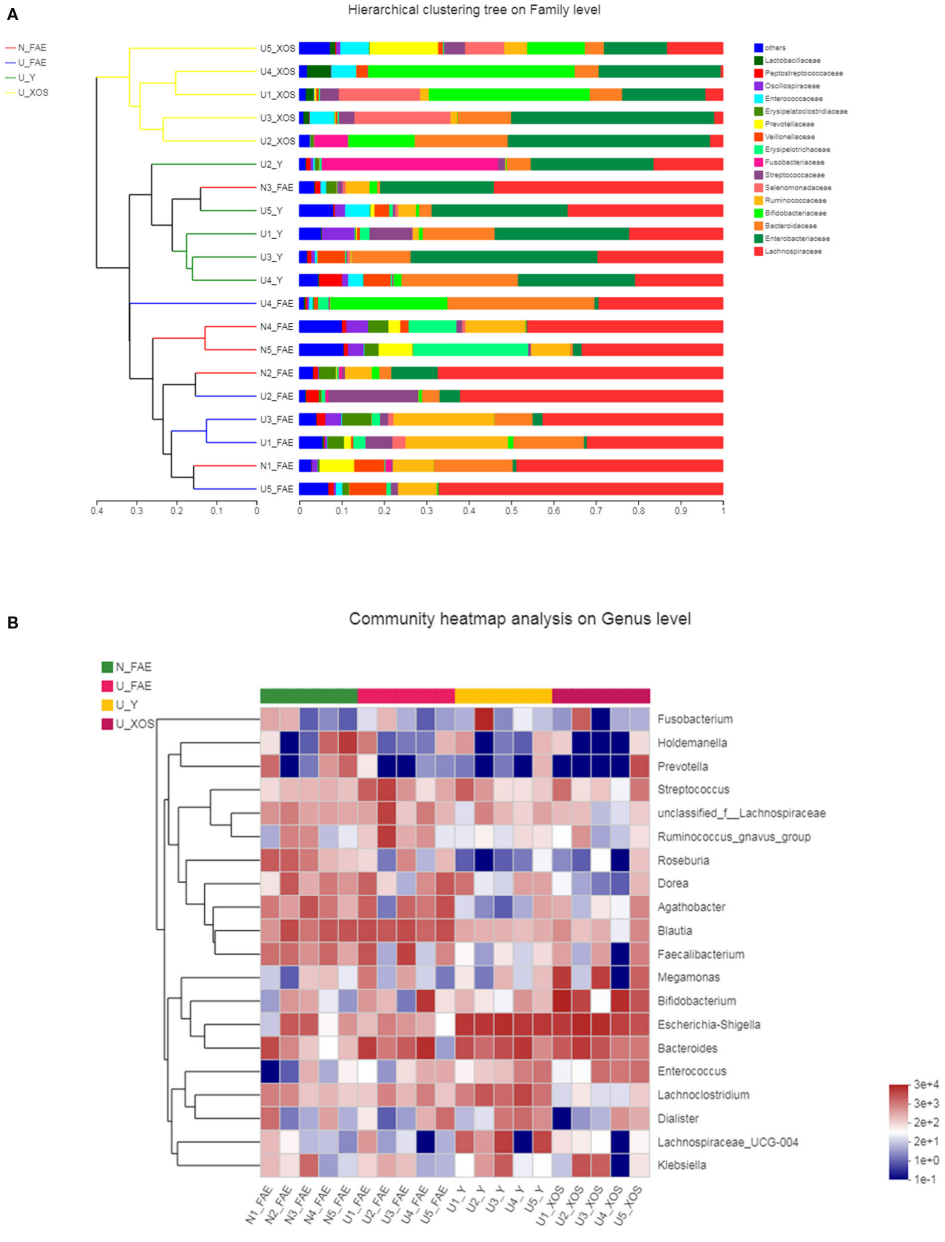
Top-species analysis of the four groups. **(A)** Hierarchical clustering tree on family level; **(B)** community heatmap analysis on genus level.

A hierarchical clustering tree was constructed to illustrate the microbiota composition and show the cluster relationship of every sample (Figure 7A). In this analysis, we found that samples of the N_FAE, U_FAE, U_Y, and U_XOS groups separated well. Community heatmap analysis at the genus level revealed the top 20 taxa of the four groups and the differentially abundant taxa, such as *g_Roseburia* and *g_Bifidobacterium*. In addition, a phylogenetic tree was drawn to illustrate the evolutionary relationship of the top 20 species via a community heatmap (Figure 7B).

## Discussion

UC is characterized by colonic inflammation, remission and relapse, the etiology and pathogenesis of this intractable disease are still unclear (1). Increasing evidence has shown that gut microbiota dysbiosis plays an important role in UC development and progression (2). In addition, the interplay between immune system disorders and gut microbiota dysbiosis has attracted the interest of researchers. New therapeutic methods, such as fecal microbiota transplantation (FMT), have been proven to be safer and more efficacious therapeutic approach for microbiota-associated diseases (18, 19).

Another potential way to modulate or maintain gut microbiota composition is the application of prebiotics, which are defined as substances that selectively stimulate the growth of beneficial microbes, particularly administered in combination with their target group of beneficial microbes (20). To date, many studies have assessed the preventive or severity-reducing effects of prebiotics in UC patients (6, 7). While some studies have shown that prebiotics are beneficial in inducing remission, others have shown no significant influence on UC. The discrepancy may be associated with disease status, individual gut microbiota differences and prebiotic type. Thus, the search for efficient prebiotics is critical.

XOS is one of the most efficient prebiotics for proliferation of *Bifidobacterium* (10), which produces a variety of organic acids and inhibits the growth of harmful bacteria via changing the gut microbiota composition. Generally, the best models for investigation of prebiotic effects on intestinal microbiota were *in vivo* testing involving use of humans and animals (21). However, *in vivo* human trials deserve strict ethical constraints and *in vivo* animal trials often give results that are not reproducible in humans (22). Furthermore, *in vivo* testing is often expensive and time consuming, usually with limited study numbers while *in vitro* fermentation models can be characterized by inoculation of single or multiple ingredients with fecal microbiota in the meantime (23).

Therefore, in the present study, we evaluated the prebiotic effect of XOS on the fecal microbiota of patients with UC in clinical remission using an *in vitro* fermentation model. The results indicated that the feces of healthy volunteers had more unique species and most species in the gut microbiota of UC patients could be cultured by a static fermentation model, which meant that this simulation model of the gut microbiota was practicable. The species richness and diversity indexes in patients with UC in remission were lower than those in healthy volunteers, but the differences were not significant, which implied that the gut microbiota may be recovered after disease remission. Moreover, the α-diversity decreased after fermentation, especially in the U_XOS group, but the difference was not significant (Figure 2). This revealed that some bacteria could not be cultured, while some were overgrown, especially with XOS fermentation, which did not significantly change the α-diversity. In general, XOS fermentation could not improve the α-diversity of the gut microbiota in patients with UC in remission. PCA showed that the four groups were obviously separated, which meant that the differences among the four groups were significant. More importantly, PCoA showed results similar to those of PCA, but the fecal samples of UC patients were more different from the fermentation samples than from N_FAE, which meant that the gut microbiota community composition was clearly changed by fermentation.

To illustrate the effects of XOS on the gut microbiota of UC patients in clinical remission, wilcoxon rank-sum test bar plots at the genus level were used, and results showed that *g_Roseburia, g_Fusicatenibacter, g_Bifidobacterium*, and *g_Lactobacillus* were more abundant, and *g_Oscillibacter, g_Bilophila*, and *g_Lachnospiraceae_UCG-010* were less abundant, in the U_XOS group than in the U_Y group.

The results of this *in-vitro* study were consistent with previously published data showing stimulation of *Bifidobacterium* enrichment by XOS (24). *Bifidobacterium*, a classic probiotic, has obvious effects on the alleviation of UC symptoms and inflammation, and is widely used in the management of UC (25–27). However, the abundances of *Bifidobacterium, Lactococcus* and *Lactobacillus* were higher (no significant) in UC patients in remission than in healthy volunteers, which may be explained by prior intake and colonization by probiotics. Moreover, *Roseburia*, a butyrate-producing bacterium, significantly decreased in patients with IBD in clinical remission and increased obviously by XOS in this study. A previous study showed *Roseburia* had an anti-inflammatory effect on dextran sulfate sodium (DSS)-induced colitis (28) and alleviated colitis by maintaining the Treg/Th17 balance in an experimental colitis model (29). Another study demonstrated that *Roseburia* flagellin inhibited activation of the NLRP3 inflammasome and pyroptosis via miR-223-3p/NLRP3 signaling in macrophages (30). All these reveal the importance of *Roseburia* in UC development and management. Furthermore, in patients with ulcerative colitis in the central European part of Russia, there was a significant decrease in the genus of *Fusicatenibacte*. The current research showed that the supplementation of XOS could increase the level of *Fusicatenibacter*, which may be beneficial for the UC treatment (31). Additionally, the number of *Lactococcus* and *Lactobacillu*s, increased under the supplementation of XOS (Figure 6). It is known that representatives of *Lactococcus* and *Lactobacillus* have therapeutic properties such as improvement of normal microbiota, prevention of infectious diseases and food allergies, modulation of innate and adaptive immune response (32). All these results indicated that XOS could promote the growth of some probiotics in the gut microbiota of UC patients in remission, which may be conducive to the alleviation of inflammation. But due to the limited sample size and lack of clinical trials, the clinical effect of XOS in UC patients still needs to be proven in the future.

In conclusion, our research indicates that this fermentation model provides a convenient *in-vitro* method for screening effective prebiotics for UC patients. The findings also suggest that XOS has the potential to relieve dysbiosis in UC patients in clinical remission, thus XOS may represent a potential prebiotic for UC management. The sample size of our study is small and further *in-vivo* studies are needed to verify the clinical effects of XOS.

## Data Availability Statement

The data presented in the study are deposited in the NCBI repository, accession number PRJNA787766.

## Ethics Statement

The studies involving human participants were reviewed and approved by Ethics Committee of Chinese PLA General Hospital (S2016-130-01). The patients/participants provided their written informed consent to participate in this study.

## Author Contributions

YY and XW: conceptualization. ZoL and ZhL: methodology, formal analysis, and writing-original draft preparation. LZ and ND: resources. GS and LP: writing-review and editing. All authors have read and agreed to the published version of the manuscript.

## Funding

This work was supported by the Ministry of Science and Technology of the People's Republic of China (2015AA020701).

## Conflict of Interest

The authors declare that the research was conducted in the absence of any commercial or financial relationships that could be construed as a potential conflict of interest.

## Publisher's Note

All claims expressed in this article are solely those of the authors and do not necessarily represent those of their affiliated organizations, or those of the publisher, the editors and the reviewers. Any product that may be evaluated in this article, or claim that may be made by its manufacturer, is not guaranteed or endorsed by the publisher.
