# Supplementary material for: Effects of Xylo-Oligosaccharide on the Gut Microbiota of Patients With Ulcerative Colitis in Clinical Remission
Source: Front Nutr. 2021 Dec 28;8:778542. doi: 10.3389/fnut.2021.778542 (PMC8748261; doi:10.3389/fnut.2021.778542)
Supplement: Supplementary file 1 [file Table_1.DOCX]

Supplementary Material

| Participant Number | Sex | Age (years) | Participant Number | Sex | Age (years) |
| --- | --- | --- | --- | --- | --- |
| U1 | male | 59 | N1 | male | 60 |
| U2 | male | 18 | N2 | male | 47 |
| U3 | female | 38 | N3 | female | 38 |
| U4 | male | 39 | N4 | male | 24 |
| U5 | male | 47 | N5 | male | 38 |

**Supplementary Figure 1** **Age and sex of UC patients and healthy volunteers.**
